# Proteomic Adaptations in the Spinal Cord of a Breast Cancer Model of Paclitaxel-Induced Peripheral Neuropathy

**DOI:** 10.1021/acs.jproteome.6c00124

**Published:** 2026-07-23

**Authors:** Samantha P. Schwarting, Virginia Espina, Paul S. Russo, Sara M. Herz, Abrar Khan, Yogesh Rakholia, Sandra Makar, Paula D. Bos, M. Imad Damaj, Nadine Kabbani

**Affiliations:** † School of Systems Biology, 3298 George Mason University, Manassas, Virginia 20110, United States; ‡ Center for Applied Proteomics and Molecular Medicine, Manassas, Virginia 20110, United States; § Department of Pharmacology & Toxicology, 6889 Virginia Commonwealth University, Richmond, Virginia 23298, United States; ∥ Department Pathology, Massey Comprehensive Cancer Center, 6886 Virginia Commonwealth University School of Medicine, Richmond, Virginia 23298, United States

**Keywords:** chemotherapy-induced peripheral neuropathy, paclitaxel, breast cancer, proteomics, drug development

## Abstract

Chemotherapy-induced peripheral neuropathy (CIPN) is a debilitating and dose-limiting side effect of taxane-based chemotherapy used in breast cancer treatment. Despite its prevalence, no FDA-approved therapies exist for CIPN prevention or treatment, highlighting the need for target prioritzation studies. Using label-free liquid chromatography–electrospray ionization tandem mass spectrometry (LC-ESI-MS/MS), we examined the effects of four doses of paclitaxel (8 mg/kg) on the lumbar (L4–L6) spinal cord proteome in MMTV-PyMT-derived breast cancer bearing C57BL/6J mice. Breast cancer altered the expression of 551 proteins relative to naïve mice, while paclitaxel treatment in tumor-bearing mice altered 112 proteins relative to vehicle-treated cancer controls. Thirty-eight proteins were commonly affected by both conditions. Gene ontology enrichment and STRING protein–protein interaction analyses identified pathways linked to mitochondrial metabolism, ion transport, and neurotransmitter signaling. Pathway mapping further revealed convergent effects of breast cancer and paclitaxel on neuronal activation, neurotransmitter uptake, and mitochondrial dysfunction. Together, these findings define spinal cord proteomic alterations associated with acute paclitaxel-induced peripheral neuropathy and identify pain-relevant pathways for future mechanistic studies.

## Graphical abstract

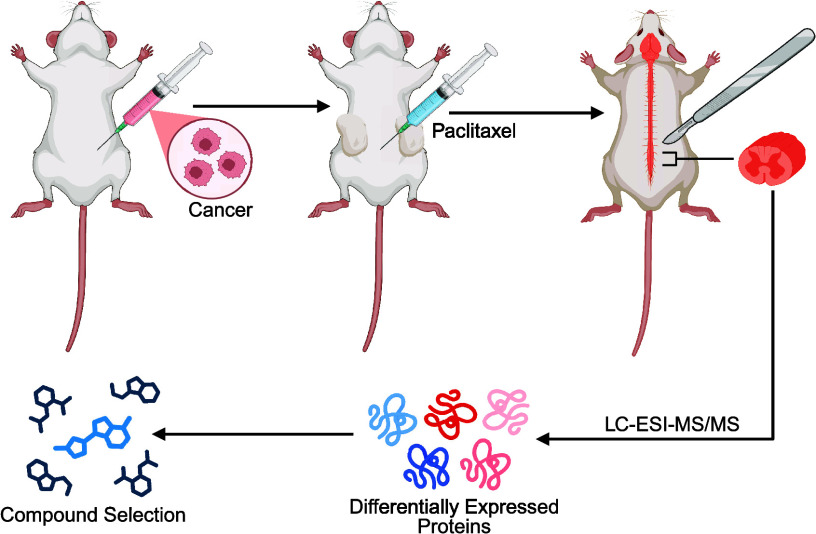

## Introduction

Chemotherapy-induced peripheral neuropathy (CIPN) is a major complication of cancer treatment, marked by sensory disturbances including numbness, tingling, neuropathic pain, and in severe cases, motor impairment.^1,2^ CIPN affects up to 70% of patients receiving taxane-, platinum-, or vinca alkaloid–based chemotherapy, with approximately 40% experiencing symptoms that persist for three months or longer.^3,4^ Among taxanes, paclitaxel remains a cornerstone of breast cancer therapy but is highly neurotoxic.^5,6^ The resulting neuropathy not only diminishes patient quality of life but often leads to dose reduction or early treatment discontinuation, ultimately compromising survival outcomes. Despite its prevalence and clinical impact, no FDA-approved therapies exist to prevent or treat CIPN, underscoring a critical gap in supportive cancer care.

Taxanes exert antitumor effects by stabilizing microtubules, including the mitotic spindle, thereby disrupting dynamic remodeling required for cell division and inducing mitotic arrest and apoptosis.^6,7^ Paclitaxel’s potent antineoplastic activity, however, is contrasted by its tendency to induce peripheral neurotoxicity through mechanisms involving mitochondrial dysfunction, impaired axonal transport, calcium dysregulation, and neuroinflammatory signaling.^8–12^ Neuroinflammatory pathways are further exacerbated in patients with breast cancer, where tumor–host interactions can disrupt neural tissues in sensory pain relevant regions, including the spinal cord.^13–16^

Despite extensive preclinical work, most of the previous studies have focused on transcriptomic analysis, which does not always correspond to protein changes and thus may not effectively translate into a therapeutic opportunity.^17–19^ Furthermore, most existing animal models of CIPN rely on naïve rodents treated with chemotherapy in the absence of cancer, limiting translational relevance by oversimplifying the complex interactions between systemic cancer–related effects and chemotherapeutic drug mechanisms. These models have revealed important mechanisms such as mitochondrial injury, axonal degeneration, and immune activation,^9,20,21^ yet they fail to account for the profound effects of tumor burden on systemic immunity, metabolic homeostasis, and chemotherapy distribution.

Several preclinical mouse models treated with paclitaxel have been shown to develop paclitaxel-induced peripheral neuropathy (PIPN).^22,23^ The mouse mammary tumor virus -Polyoma Virus middle T antigen (MMTV-PyMT) model is a breast cancer model shown to recapitulate stepwise tumor progression including systemic immunological responses^24–26^ and has been used to determine the antitumor efficacy of paclitaxel.^27–30^ Here, we used the MMTV-PyMT model to examine the impact of paclitaxel on proteomic remodeling within the spinal cord, an anatomical region tightly linked to sensory hypersensitivity and pain processing during PIPN.^31–33^ Our proteomic analyses reveal paclitaxel-associated adaptations that may contribute to PIPN in the context of breast cancer and support a translational framework for future studies aimed at improving the treatment of PIPN.

## Methods

### Animals

The experiments were performed on 10 week-old female C57BL/6J mice (Strain #000664, The Jackson Laboratory, Bar Harbor, ME). Animals were housed in groups of 4 per cage with an enriched environment and maintained on a 12 h light/dark cycle, at a 22 °C room temperature with ad libitum access to food (global 18% protein chow diet; Envigo Teklad, Indianapolis, IN, USA) and water. All animal experiments were performed under an approved IACUC protocol (VCU AM-10142) in the Division of Animal Research of Virginia Commonwealth University (Richmond, VA), accredited by the Association for Assessment and Accreditation of Laboratory Animal Care (AALAC). All efforts were made to reduce the number of animals used in this study and to ensure optimal conditions of well-being before, during, and after each experiment. All mice were observed daily for general well-being, and their weight was measured weekly.

### MMTV-PyMT Breast Tumor Model

Female MMTV-PyMT mice were monitored for spontaneous tumor formation, and tumors were harvested when they reached a volume of 1000–1500 mm.^3^ Tissues were minced and enzymatically digested using 400 μg/mL Liberase TL (Roche) in a rotary shaker for 30 min at 37 °C. Single cells were obtained by filtration through 100 μm cell strainers (Fisherbrand) and were centrifugated at 300*g* for 5 min. 150,000 tumor cells were resuspended in PBS and mixed at a 1:1 ratio with growth factor reduced Matrigel (BD). The cell suspension was injected bilaterally into the fourth mammary gland of isoflurane-anesthetized mice. Primary tumor growth was monitored three times a week by caliper measurements of tumor length (*L*) and width (*W*). Tumor volume was calculated using the formula (*L* × *W* ^2^) ÷ 2.

### Drug Treatment

Paclitaxel was purchased from VCU Health Pharmacy (Athenex, NDC 70860-200-50, Richmond, VA, USA) and dissolved in a 1:1:18 mixture of 200 proof ethanol, kolliphor, and distilled water (Sigma-Aldrich). Twelve days after tumor implantation when tumors reached 100–150 mm^3^ in volume, paclitaxel was administered at a dose of 8 mg/kg intraperitoneally (i.p.) every other day; 4 administrations completed one regimen. Control mice received an equal volume injection of the vehicle, with the same regimen. This resulted in a well-characterized paclitaxel CIPN mouse model as described by our group.^8,23^ Seven days after the first dose of drug treatment, mice were euthanized by cervical dislocation. Lumbar (L4-L6) spinal cord tissues from both experimental and control groups were collected, immediately frozen in liquid nitrogen, and stored at −80 °C.

### Spinal Cord Protein Isolation

Frozen spinal cord tissue specimens were solubilized in Rapigest SF Surfactant (1 mg/mL in 50 mM Ammonium Bicarbonate) (Waters 186001860) with 5 mM dithiothreitol, vortexed on ice, and homogenized using a tube pestle. Chicken lysozyme (Sigma L4919, 300 ng/μL) was added to each spinal cord homogenate. Samples were incubated for 30 min at 60 °C, cooled to room temperature, and spun in a centrifuge at 4 °C for 5 min at 16.1 rcf. Total protein was measured using a protein 660 nm assay (Pierce 2262) per manufacturer’s instructions. 20 μg of protein from each sample was alkylated using 50 mM iodoacetamide at room temperature in the dark for 20 min and digested with trypsin (4 μL, 0.1 μg/μL) (Promega, V511B) in 50 mM Ammonium Bicarbonate at 37 °C. Digestion was stopped by adding 2 μL of 100% trifluoracetic acid (TFA) and the samples were heated at 37 °C for 30 min, spun for 10 min at 16.1 rcf, and the supernatants were desalted with C-18 spin columns (Pierce, #89870). Proteins were eluted in 80% acetonitrile in water with 0.1% trifluoroacetic acid (Riedel-deHaen 34976). Final eluates were dried using a SpeedVac vacuum concentrator (ThermoFisher, Waltham, MA, USA). Samples were reconstituted in 10 μL of 0.1% formic acid in water.

### Liquid-Chromatography Electrospray Ionization Mass Spectrometry

LC-ESI-MS/MS experiments were performed on an Orbitrap Exploris 480 (ThermoFisher Scientific) equipped with a nanospray EASY-nLC 1200 HPLC system (Thermo Fisher Scientific). Peptides were separated using a PepMap RSLC 75 μm i.d. × 20 mm long with 3 μm, C18 resin trap column (#164946, ThermoFisher Scientific) and a EASY-Spray HPLC column (75 μm i.d. x 150 mm long, packed with 2 μm ultrapure silica (#03-251-875, Fisher Scientific). The mobile phase consisted of 0.1% aqueous formic acid (mobile phase A) and 0.1% formic acid in 80% acetonitrile (mobile phase B). After sample injection, the peptides were eluted by using a linear gradient from 5% to 50% B over 90 min and ramping to 100% B for an additional 2 min. The flow rate was set at 300 nL/min. The Exploris 480 was configured to acquire data using data independent analysis (DIA) with 60 × 10 *m*/*z* MS/MS isolation windows (window overlap of 1 *m*/*z*, range of 385–975 *m*/*z*, S-Lens RF 70%, 15,000 resolution, normalized AGC target of 200%, max IT of 40 ms and NCE of 27). Precursor spectra (range of 380–985 *m*/*z*, S-Lens RF 40%, 60,000 resolution, normalized AGC target of 100%, max IT of 100 ms) were included in between every 30 MS/MS spectra. For all experiments, the precursor data were collected in profile mode and the MS/MS data were collected in centroid mode.

### Statistical Analysis

DIA raw data files were converted to HTRMS format using HTRMS Converter v19 and analyzed with Spectronaut v19.9 (Biognosys, Switzerland). Protein identification was performed against the *Mus musculus* UniProtKB reviewed database (August 2025 release; 17,855 entries) using default Spectronaut settings. Methionine oxidation and N-terminal acetylation were included as variable modifications, while cysteine carbamidomethylation was specified as a fixed modification. Trypsin specificity was applied with a maximum of two missed cleavages allowed. Peptide and protein identifications were filtered at a 1% false discovery rate (FDR). Label-free quantification was performed at the MS2 level using area-under-the-curve integration, and cross-run normalization was carried out using the default local normalization strategy (Runs <500).

Proteins were filtered to retain those with nonmissing values in at least 60% of samples within each condition. Protein abundance values were log2-transformed prior to statistical analysis to better approximate a normal distribution. Differential abundance testing was performed using Welch’s *t*-test with Benjamini–Hochberg correction and a significance threshold of adjusted *p* < 0.05. Pairwise comparisons included naïve vs. MMTV-PyMT mice (*n* = 8 vs. 3), MMTV-PyMT vs. paclitaxel-treated MMTV-PyMT mice (*n* = 3 vs. 5), and naïve vs. paclitaxel-treated naïve mice (*n* = 8 vs. 8).

### Bioinformatics

Gene ontology (GO), KEGG, Reactome, and WikiPathways enrichment analyses were conducted using the Search Tool for the Retrieval of Interacting Genes/Proteins (STRING, v12.0) database.^34^ Protein clustering was also performed in STRING using a Markov Cluster Algorithm (MCL) with an inflation parameter of 3 and depicted with the Cytoscape STRINGApp^35,36^ ^.^Candidate compounds for CIPN prevention or treatment were identified using OpenTargets’ ChEMBL annotation.^37,38^ Data was processed, analyzed, and presented using Excel and the following tools: the R statistical software^39^ and packages: dplyr,^40^ stringr,^41^ ggplot2,^42^ gplots,^43^ pheatmap,^44^ tidyr,^45^ broom,^46^ purrr,^47^ readxl,^48^ and writexl.^49^

## Results

### Proteomic Analysis of Spinal Cord in a Mouse Model of Breast Cancer

As shown in Figure 1, we tested the effect of paclitaxel administration (4 injections at 8 mg/kg every other day) and cancer status (tumor-bearing vs. naïve) on proteins within spinal cord lumbar region (L4-L6) using high throughput LC-ESI MS/MS in DIA mode. This analysis enables the identification of global protein changes between cancer-bearing and naïve, as well as paclitaxel and vehicle treatment conditions. With this regimen, paclitaxel treatment effectively reduces tumor volume, consistent with its chemotherapeutic efficacy (Figure S1), while also promoting the pain sensitivity associated with CIPN, as previously described by our group.^8,23^

**Figure 1:**
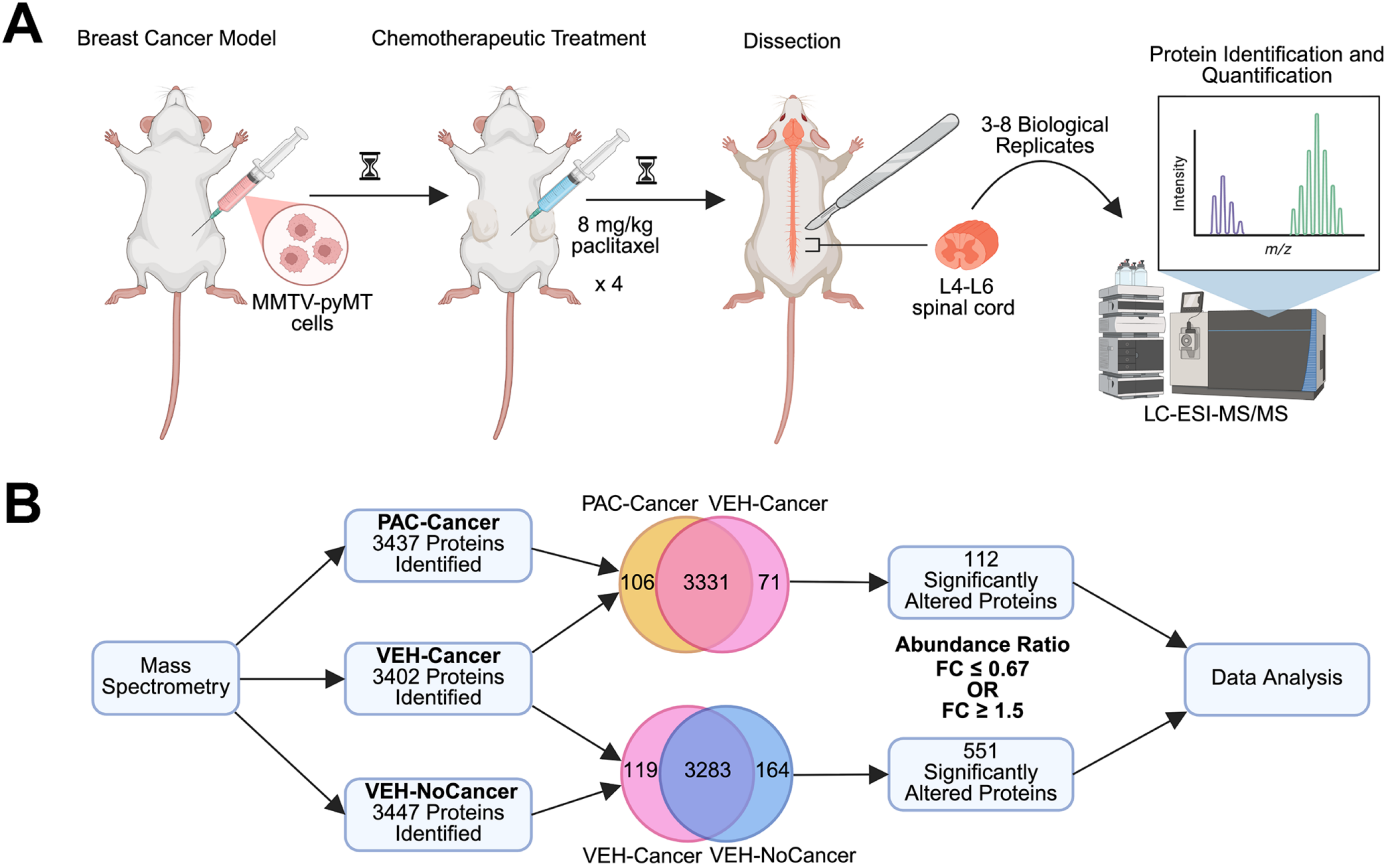
Workflow to identify proteomic alterations in a CIPN model. (A) Experimental design. MMTV-PyMT cells were injected into mice to establish an orthotopic, syngeneic breast cancer model. Following tumor development, naïve or tumor-bearing mice were treated with a clinically relevant dose of paclitaxel (8 mg/kg) or a vehicle control. The L4-L6 spinal cord was dissected for LC-ESI-MS/MS proteomic analysis. (B) Proteomic analysis pipeline and overview. Created with Biorender (Schwarting, S.P., 2026; https://BioRender.com/ni5gxih).

As shown in Figure 1B, we identified unique protein groups based on peptide spectral matches (PSMs) in the L4-L6 spinal cord tissue of paclitaxel-treated MMTV-PyMT (PAC-Cancer), vehicle-treated MMTV-PyMT mice (VEH-Cancer), and naïve (cancer-free) mice (VEH-NoCancer). Analysis of proteome using principal component analysis (PCA) shows the segregation of biological replicates by treatment (paclitaxel vs. vehicle) and cancer status (cancer-bearing vs. naïve) (Figure S2). These results indicate an effect of the experimental conditions on spinal cord proteome. We detected 3437 proteins within the paclitaxel-treated cancer-bearing proteome and 3402 proteins within the vehicle-treated cancer-bearing proteome, with 3331 (95.0%) shared proteins between the two groups (Figure 1B). We also conducted quantitative proteomic analysis comparing the spinal cord proteome between tumor-bearing and nontumor-bearing vehicle-treated mice. As shown in Figure 1B, among the 3402 proteins detected within the spinal cord of tumor-bearing mice, approximately 3283 (92.1%) proteins were shared with the 3447 proteins detected within the spinal cord of the nontumor-bearing mice.

To identify the effects of paclitaxel and cancer on the protein expression of the spinal cord, we compared the changes in protein abundance (abundance ratio) using the criteria of ≤0.67 (−33%) and ≥1.5 (+50%) change in the protein’s abundance ratio. These analyses provide insight into proteome-wide modification within the spinal cord in response to the cancer and paclitaxel experimental conditions.

### The Effect of Breast Cancer on Spinal Cord Proteomes

Breast cancer exerts systemic effects in mouse models that mimic human disease including the activation of immune signaling and neuroinflammation.^50–52^ We examined the effect of breast cancer on spinal cord protein expression within MMTV-PyMT tumor-bearing and naïve mice (VEH-Cancer vs. VEH-NoCancer). A total of 3402 and 3447 proteins were identified within the spinal cord of tumor-bearing and naïve mice, respectively. Statistical analysis indicates an effect of cancer on 551 unique proteins between cancer-bearing and naïve mice (*p*_adj_ < 0.05). Specifically, we found that the presence of cancer resulted in 455 upregulated and 96 downregulated proteins (Figure 2A and Table S1). Gene ontology (GO) enrichment analysis of significantly altered proteins was conducted using STRING software. As shown in Figure 2B–D, cancer occurrence was associated with significant changes in cytoskeletal regulation, vesicular dynamics, and mitochondrial ATP synthesis.

**Figure 2:**
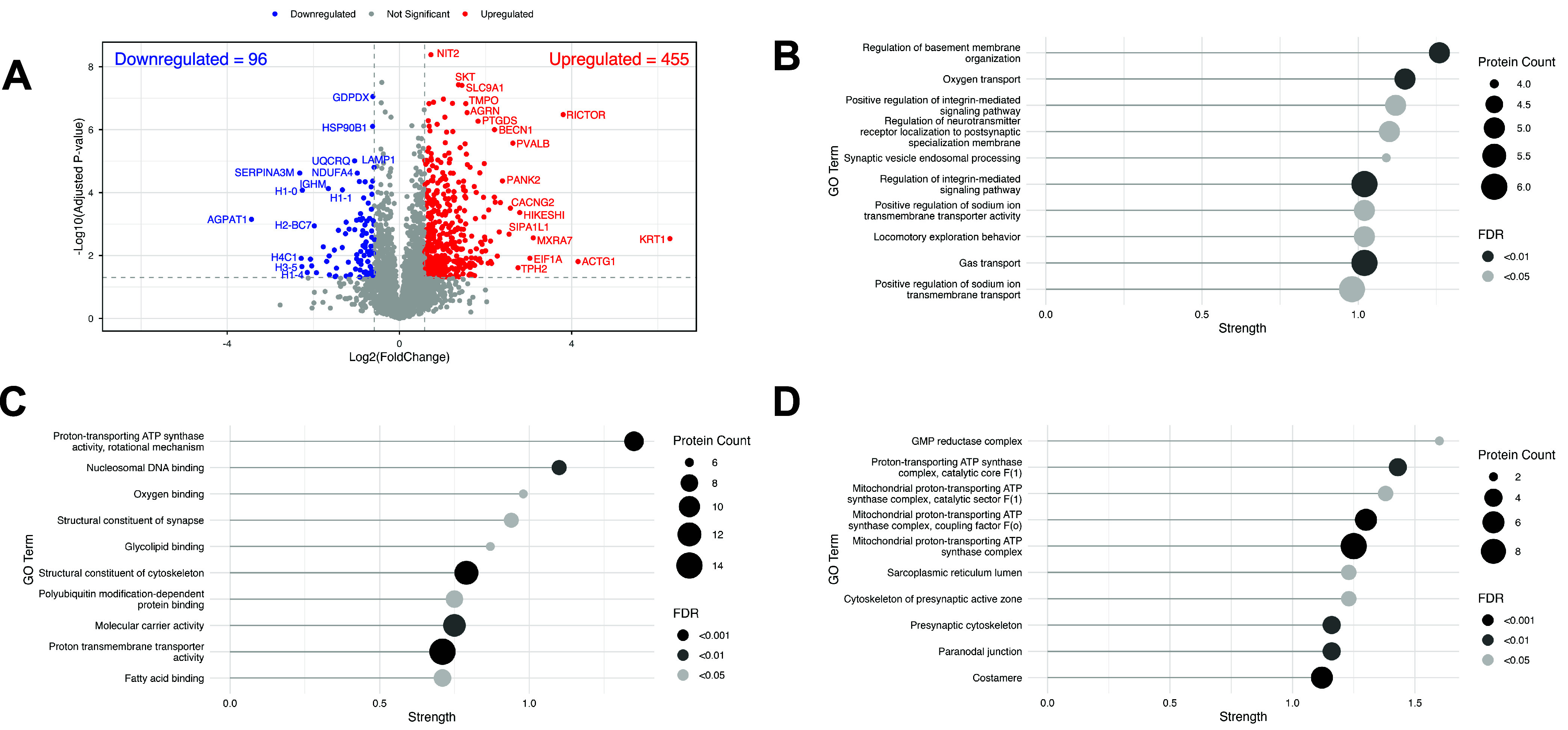
Introduction of breast cancer into mice alters the spinal cord proteome. (A) Volcano plot identifies 455 upregulated (red, ≥50% increase) and 96 downregulated (blue, ≥33% decrease) proteins in tumor-bearing mice compared to cancer-free controls (p_adj_ < 0.05). GO enrichment analysis using STRING demonstrated significantly altered proteins are enriched in (B) biological processes, (C) molecular functions, and (D) cellular components. Length along the horizontal axis represents the enrichment score assigned to the term.

We further examined the biological pathways represented in the cancer-driven upregulated and downregulated proteins in the spinal cord using KEGG, Reactome, and WikiPathways enrichment tools. As shown in Figure 3A, tumor-bearing mice exhibit an upregulation in proteins associated with RHO-GTPase signaling, cytoskeletal remodeling, and cell-extracellular matrix (ECM) interactions. Based on their known function, these protein changes suggest modifications to the cellular matrix and potentially the blood-spinal cord barrier (BSCB) in these mice.^53,54^

**Figure 3:**
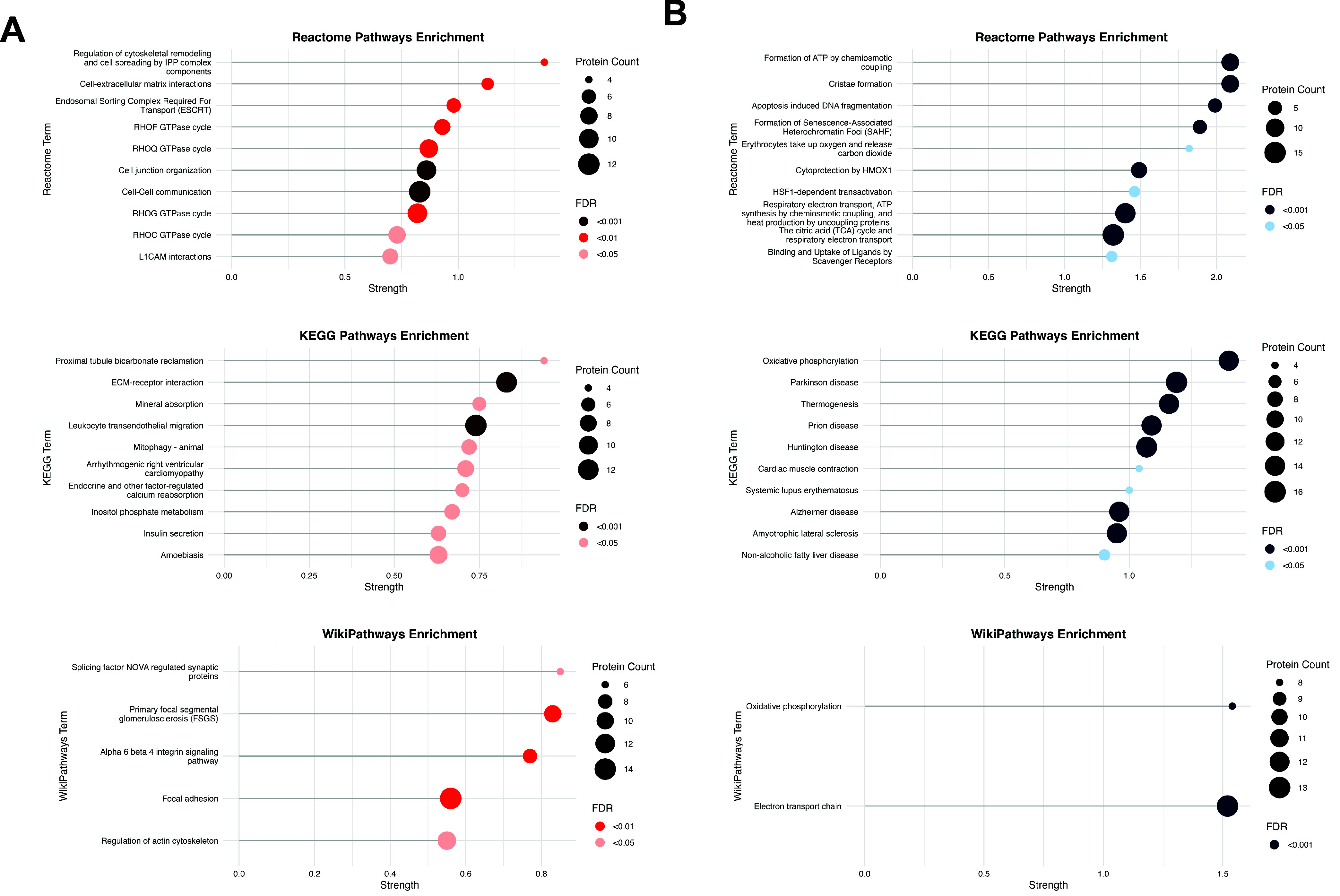
STRING pathway enrichment analysis emphasizes changes in cell organization in tumor-bearing mice relative to nontumor-bearing controls. (A) Upregulated and (B) downregulated proteins showed significant enrichment across Reactome, KEGG, and WikiPathways. Length along the horizontal axis represents the pathway enrichment score.

In comparison, KEGG, Reactome, and WikiPathways analysis of downregulated proteins revealed an enrichment in oxidative phosphorylation (OXPHOS), mitochondrial structural regulation (e.g., cristae formation), and the electron transport chain (ETC) (Figure 3B). Interestingly, KEGG pathway enrichment analysis indicates an effect of cancer on OXPHOS, which is also linked to neurodegenerative disease including Parkinson’s and Huntington Disease.^55^ Taken together, these results demonstrate that breast cancer occurrence impacts the proteome of spinal cord tissue with implications for chronic pain and neuropathy.^33,56^

### Paclitaxel Treatment of Breast Cancer-Bearing Mice Impacts Spinal Cord Proteomes

We next examined the effect of paclitaxel on the spinal cord proteome in tumor-bearing mice. Proteomic analysis was conducted between paclitaxel- and vehicle-treated MMTV-PyMT tumor-bearing mice (PAC-Cancer vs. VEH-Cancer). A total of 3437 and 3402 proteins were identified within paclitaxel- and vehicle-treated spinal cord tissue samples, respectively. Statistical analysis revealed that paclitaxel treatment affected 112 spinal cord proteins between the two groups, with 85 upregulated and 27 downregulated (Figure 4A and Table S2). GO enrichment analysis of significantly altered proteins was conducted using STRING. As shown in Figure 4B–D, paclitaxel treatment was associated with changes in proteins involved in ion and amino acid transport across membranes.

**Figure 4:**
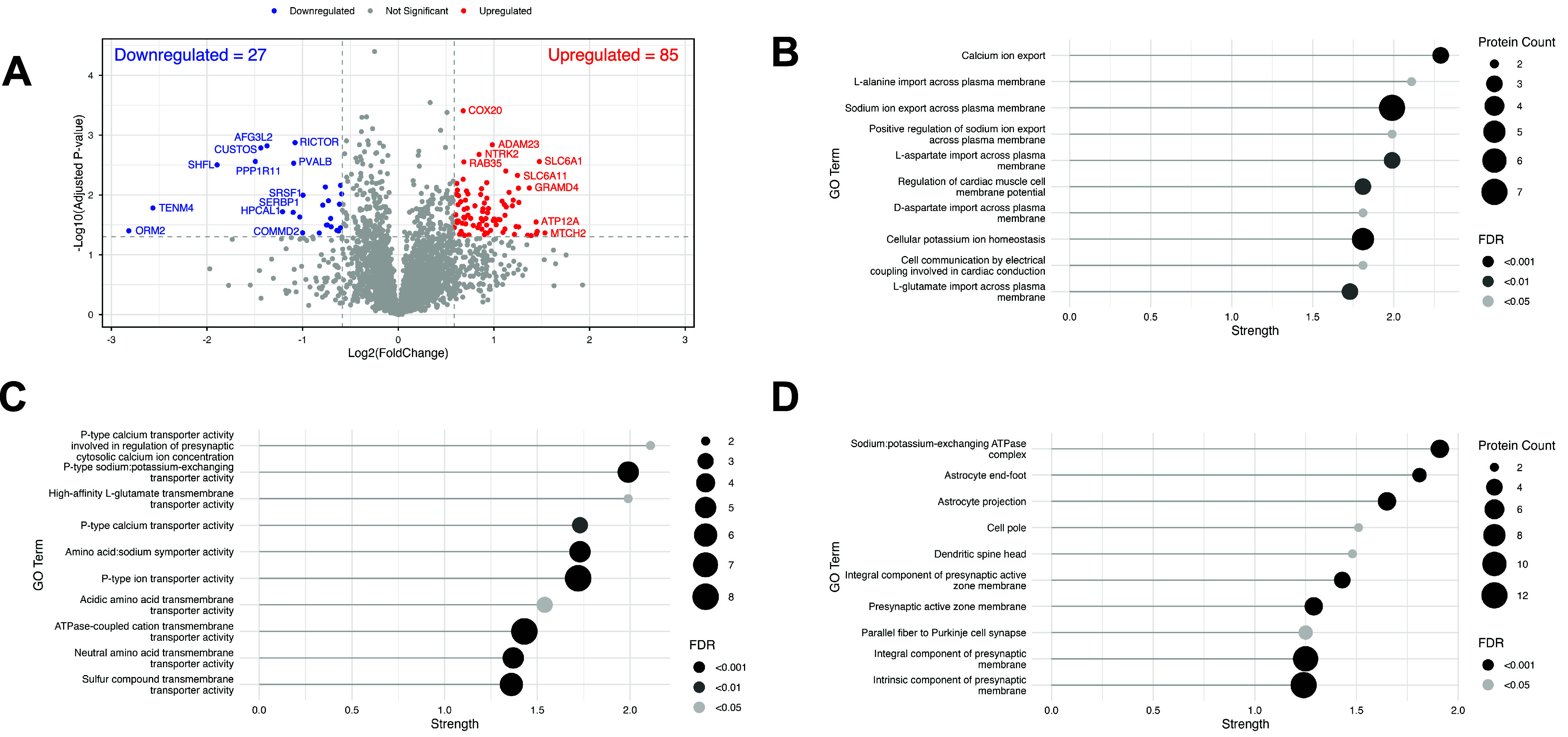
Paclitaxel treatment of tumor-bearing mice highlights proteins involved in development of CIPN. (A) Volcano plot identifies 85 upregulated (red, ≥50% increase) and 27 downregulated (blue, ≥33% decrease) proteins in paclitaxel-treated tumor-bearing mice compared to vehicle-treated tumor-bearing mice (p_adj_ < 0.05). GO enrichment analysis using STRING demonstrated significantly altered proteins are enriched in (B) biological processes, (C) molecular functions, and (D) cellular components. Length along the horizontal axis represents the enrichment score assigned to term.

GO enrichment was performed to analyze the upregulated and downregulated protein data sets. As shown in Figure 5A, STRING analysis revealed that upregulated proteins were enriched in neurotransmitter and ion transport in Reactome, osmotic processes in KEGG, and mitochondrial metabolic pathways and neuronal activity in WikiPathways. The downregulated proteins only showed enrichment in Reactome for platelet degranulation, potentially due to a limited number (27) of significantly downregulated proteins (Figure 5B). An analysis of proteins significantly altered by cancer (VEH-Cancer vs. VEH-NoCancer) and by paclitaxel treatment (PAC-Cancer vs. VEH-Cancer) identified a convergence of the two proteomes onto 38 shared proteins (Figure 5C and Table S3).

**Figure 5:**
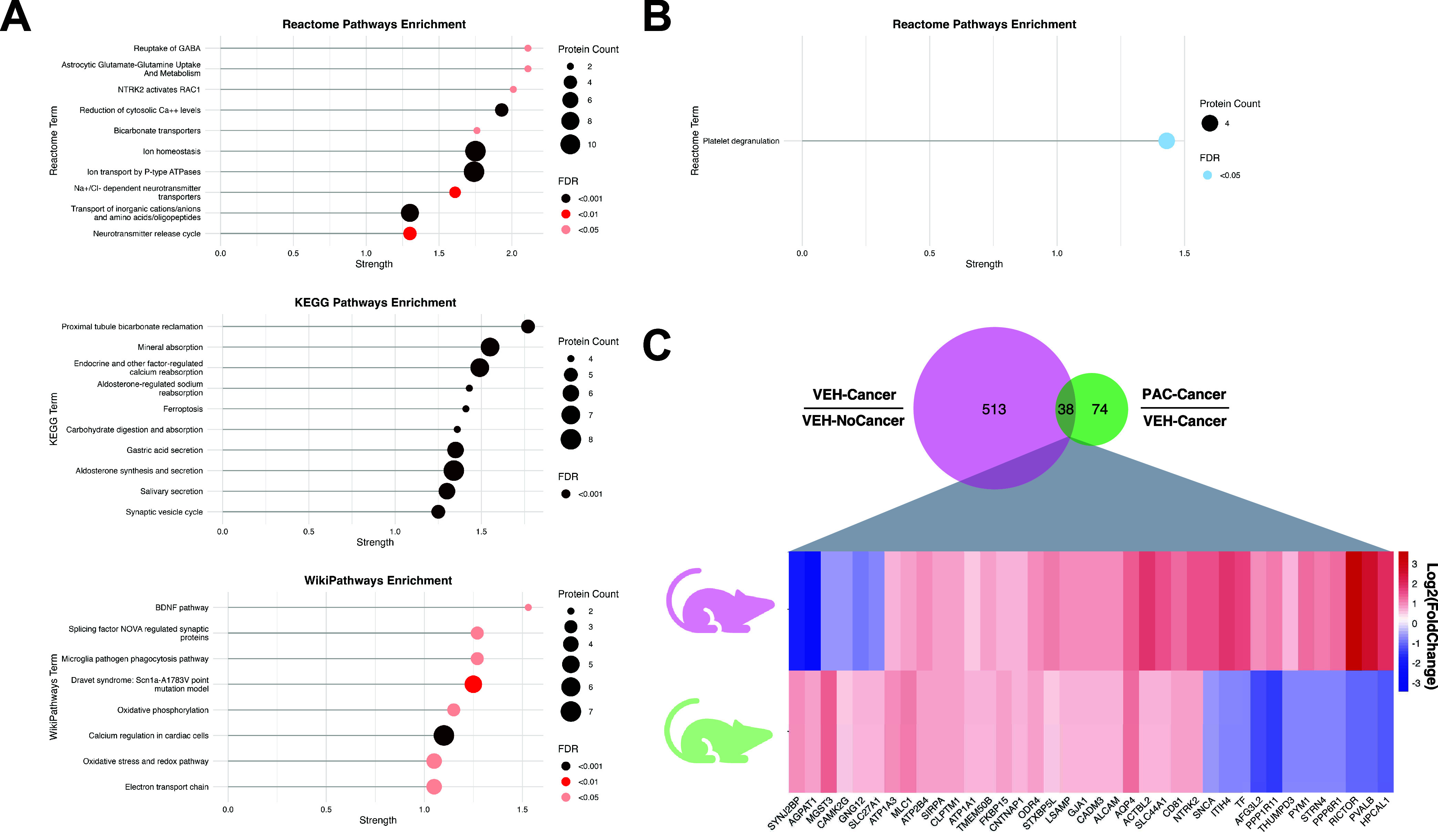
STRING pathway enrichment analysis emphasizes significant upregulation of neurotransmitter- and ion-transporting pathways in paclitaxel-treated tumor-bearing mice relative to vehicle-treated controls. (A) Upregulated proteins showed significant enrichment across Reactome, KEGG, and WikiPathways. (B) Downregulated proteins only exhibited significant enrichment in Reactome. Length along the horizontal axis represents the pathway enrichment score. (C) Venn diagram illustrating the overlap of the significantly altered proteins from the VEH-Cancer to VEH-NoCancer (pink) and the PAC-Cancer to VEH-Cancer comparison (green). Heatmap highlights log_2_ fold-changes of proteins overlapping between both comparisons.

To compare the impact of paclitaxel on spinal cord proteomes in tumor-bearing and tumor-free mice, we analyzed paclitaxel-induced proteomic changes in tumor-bearing mice (PAC-Cancer vs. VEH-Cancer) and naïve mice (PAC-NoCancer vs. VEH-NoCancer, Table S4). Paclitaxel treatment in tumor-bearing mice and naïve mice did indicate an overlap in significantly altered proteins.

We used a Markov cluster (MCL) analysis to identify pathways and interactions among significantly altered protein within paclitaxel-treated tumor-bearing mice relative to vehicle-treated control. MCL analysis identified 5 high confidence clusters associated with paclitaxel treatment (Figure 6A and Table 1). The results are consistent with previous analyses highlighting the impact of paclitaxel on mitochondrial, ion, and synaptic regulatory mechanisms within the spinal cord and sensory ganglia.^9,12,33^ Specifically, Cluster 1 indicates strong enrichment in amino acid transport components and consists of 9 proteins that are integrated within a 22 protein–protein interaction (PPI) network (*p* < 1.0 × 10^–16^). Cluster 2 contains 8 proteins and 13 PPIs involved in mitochondrial calcium transport (*p* < 1.0 × 10^–16^). Cluster 3 consists of 7 proteins that are components of the sodium–potassium pump (*p* < 1.0 × 10^–16^). Cluster 4 (*p* = 1.920 × 10^–13^) and cluster 5 (*p* = 2.26 × 10^–12^) consist of proteins involved in mitochondrial complex 1 and acute phase pathways, respectively.

**Figure 6:**
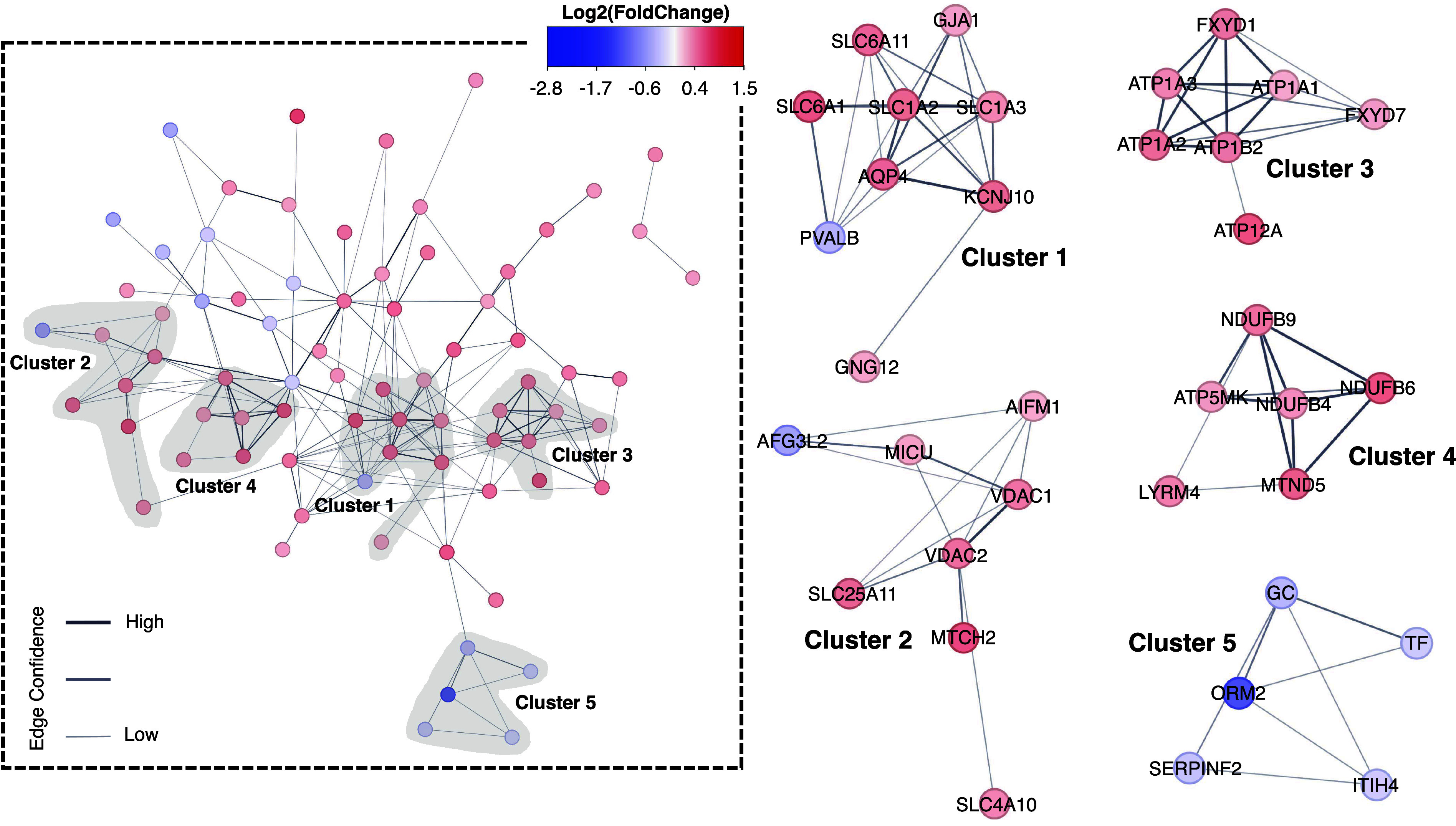
STRING protein–protein interaction (PPI) network validates neurotransmitter and ion transport alterations and highlights mitochondrial changes in paclitaxel-treated tumor-bearing mice relative to vehicle-treated controls. MCL clustering of significantly altered proteins identified the top five clusters enriched in transport of molecules, mitochondrial functions, and acute phase interactions.

**Table 1:** Top 5 Protein–Protein Interaction (PPI) Network Clusters Derived from Differentially Expressed Proteins in Spinal Cord of Paclitaxel-Treated MMTV-PyMT Breast Tumor-Bearing Mice Compared to Vehicle-Treated Controls^a^

Cluster	Description	# of nodes	# of edges	Local clustering coefficient	PPI enrichment P-value	Proteins
1	amino acid transport & amino acid: sodium symport activity	9	22	0.826	<1.0 × 10^–16^	AQP4, GJA1, GNG12, KCNJ10, PVALB, SLC1A2, SLC1A3, SLC6A1, SLC6A11
2	mitochondrial calcium ion transmembrane transport & porins	8	13	0.733	<1.0 × 10^–16^	AFG3L2, AIFM1, MICU1, MTCH2, SLC25A11, SLC4A10, VDAC1, VDAC2
3	ion transport by P-type ATPases & sodium ion transport across plasma membrane	7	16	0.952	<1.0 × 10^–16^	ATP12A, ATP1A1, ATP1A2, ATP1A3, ATP1B2, FXYD1, FXYD7
4	mitochondrial complex I assembly	6	11	0.822	1.92 × 10^–13^	ATP5MD, LYRM4, MT-ND5, NDUFB4, NDUFB6, NDUFB9
5	acute phase	5	7	0.767	2.26 × 10^–12^	GC, ITIH4, ORM2, SERPINF2, TRF

a Network analysis identified functional modules (Clusters 1–5), with annotations based on overrepresented Reactome/GO pathway terms. columns indicate cluster rank, functional description, number of nodes (proteins) and edges (interactions) in each subnetwork, average local clustering coefficient, PPI enrichment p-value (string), and the list of proteins contained in each cluster.

### Delineation of Mitochondrial Pathways Associated Paclitaxel Treatment

Our findings reveal an effect of breast cancer and paclitaxel treatment on common proteins within the spinal cord. As shown in Figure 5D and Table S3, 112 proteins were identified to be impacted by paclitaxel (PAC-Cancer vs. VEH-Cancer) with a subset of 38 proteins found to also be significantly altered in the cancer group comparison (VEH-Cancer vs. VEH-NoCancer). We hypothesize that these 38 overlapping proteins represent potentially druggable pathways relevant to CIPN during breast cancer therapy, as they reflect points of convergence between breast cancer- and paclitaxel-associated proteomic adaptations. Insights from these 38 proteins, together with functionally connected comparison-specific proteins, were incorporated into a comprehensive signaling and metabolic pathway map within a WikiPathways model (Figure 7). This theoretical pathway depicts the involvement of individual proteins and their relative expression changes within glutamatergic and GABAergic transmission pathways in the spinal cord. As illustrated, our proteomic findings suggest an effect of paclitaxel treatment, during breast cancer, on membrane sodium and potassium transport important for the regulation of neuronal firing. Moreover, our proteomic results suggest an upregulation in proteins involved in the clearance of spinal cord neurotransmitters (e.g., GABA). Additionally, paclitaxel treatment is found to modify the expression of proteins that are components of the ETC and thus ATP generation. Together, our analyses shows points of protein adaptation that may contribute to disrupted neurotransmission during CIPN.^57,58^

**Figure 7:**
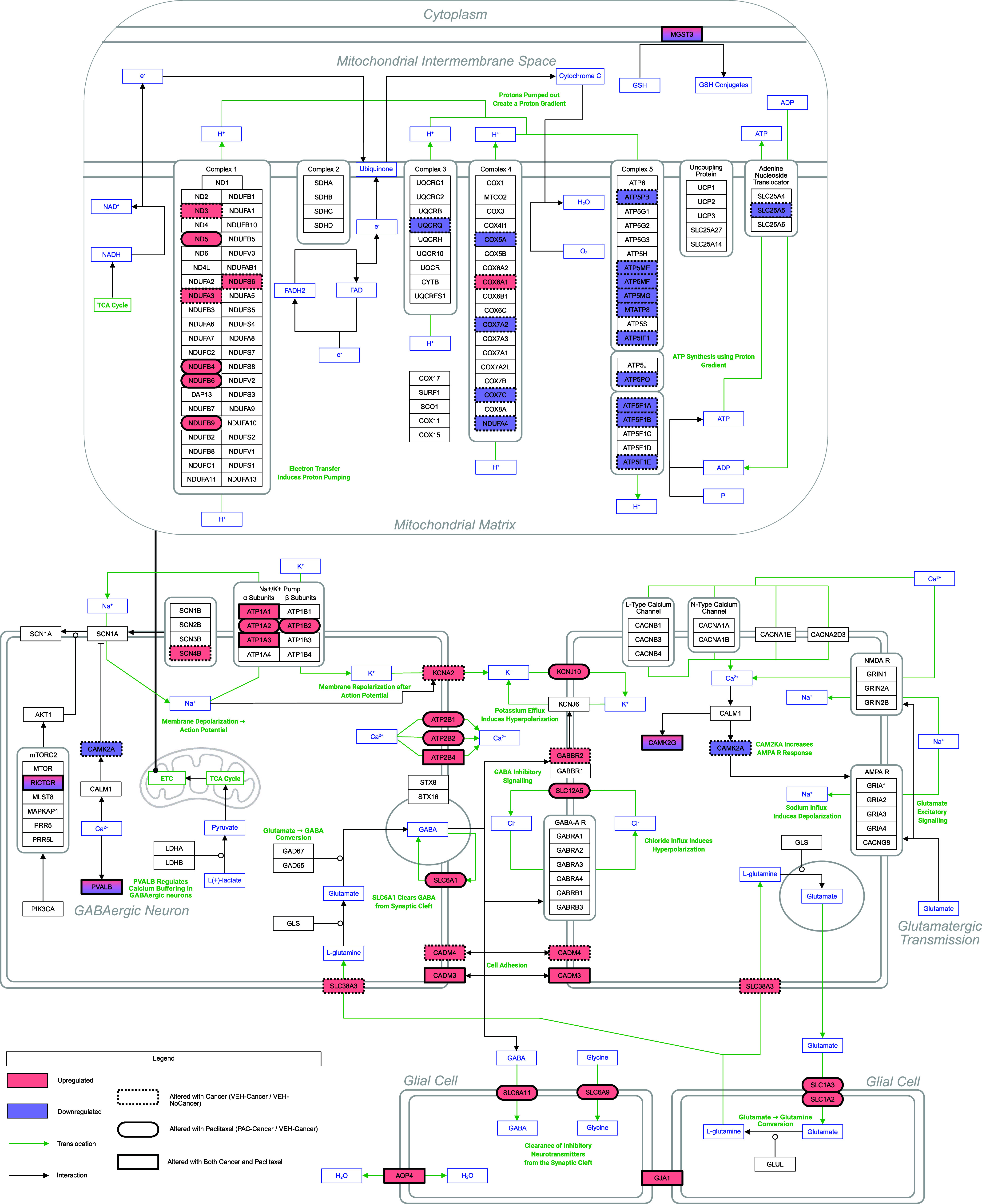
Cancer and paclitaxel alter proteins involved in GABAergic and Glutamatergic signaling. Pathway map informed by significantly altered proteins and existing WikiPathways representations of GABAergic and glutamatergic signaling interactions. Created in Biorender (Schwarting, S.P., 2026; https://BioRender.com/llmxoml).

We used OpenTargets and ChEMBL annotation to identify compounds with reported selectivity for proteins significantly upregulated in the spinal cord of paclitaxel-treated tumor-bearing mice relative to vehicle-treated tumor-bearing controls^37,38^ (Figure 8). This presents a hypothetical target discovery application based on our proteomic data, highlighting potentially druggable pathways and candidate molecular targets relevant to PIPN (Table 2).

**Figure 8:**
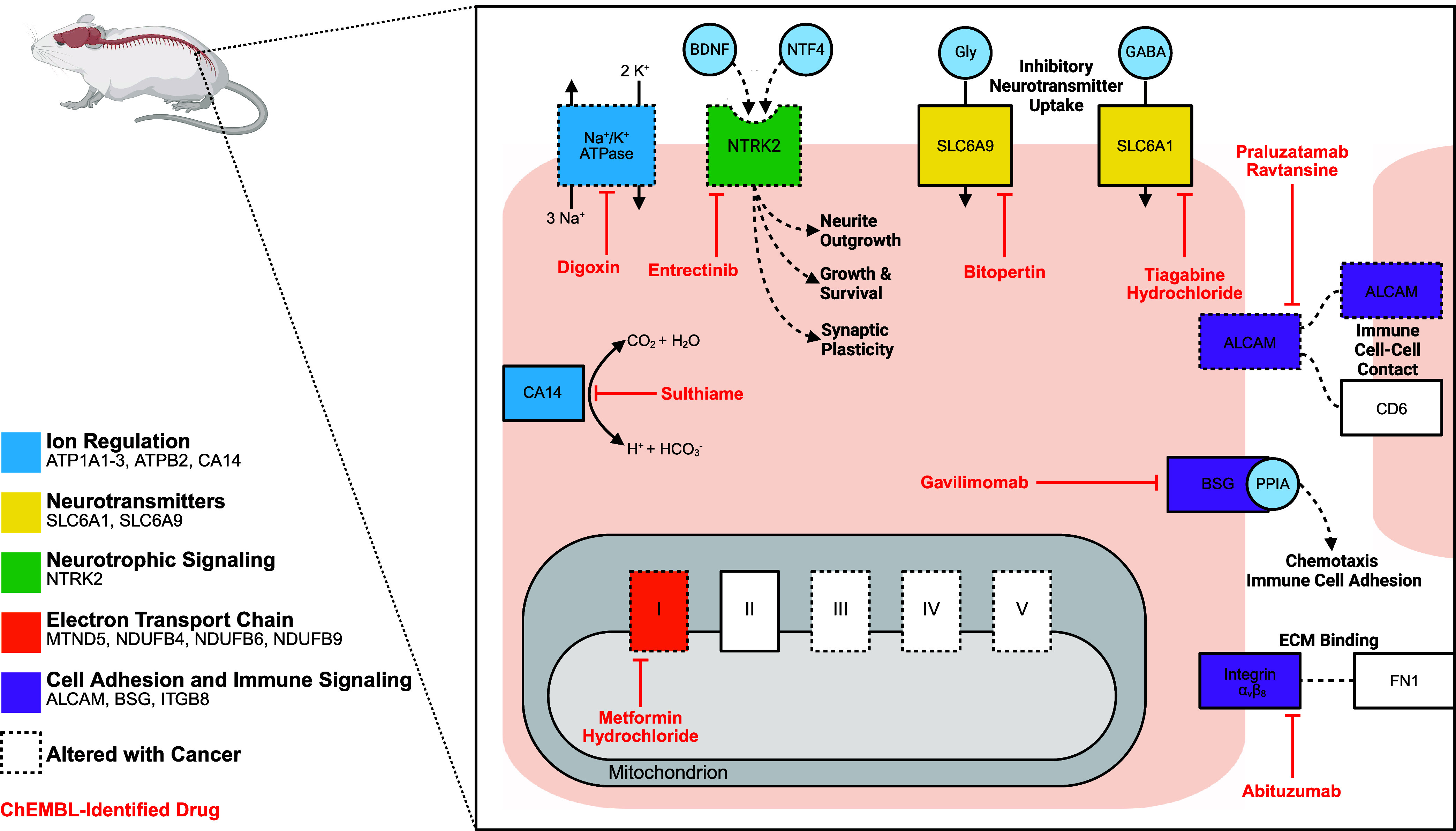
Schematic model of the available compounds for the top upregulated proteins in paclitaxel-treated tumor-bearing mice. Model summarizes compound–targets interaction with potential efficacy in PIPN treatment or prevention. Created with Biorender (Schwarting, S.P., 2026; https://BioRender.com/x5t21rp).

**Table 2:** Pharmacologically Actionable Proteins Significantly Upregulated in Lumbar Spinal Cord of Paclitaxel-Treated MMTV-PyMT Tumor-Bearing mice^a^

UniProt	Protein	Log_2_FC	ChEMBL identified drug	Mechanism of action
P31648	SLC6A1	1.46	Tiagibine Hydrochloride	GABA transporter inhibitor
Q3UIU2	NDUFB6	1.44	Metformin Hydrochloride	mitochondrial complex 1 inhibitor, NADH/ubiquinone oxidoreductase
P03921	MTND5	1.35	Metformin Hydrochloride	mitochondrial complex 1 inhibitor, NADH/ubiquinone oxidoreductase
Q6PIE5	ATP1A2	1.16	Digoxin	sodium/potassium-transporting ATPase inhibitor
P28571	SLC6A9	1.11	Bitopertin	glycine transporter 1 inhibitor
Q9CQJ8	NDUFB9	1.09	Metformin Hydrochloride	mitochondrial complex 1 inhibitor, NADH/ubiquinone oxidoreductase
P14231	ATP1B2	1.09	Digoxin	sodium/potassium-transporting ATPase inhibitor
Q0VBD0	ITGB8	0.93	Abituzumab	integrin Alpha-V/Beta-8 inhibitor
Q9WVT6	CA14	0.92	Sulthiame	carbonic anhydrase inhibitor
Q6PIC6	ATP1A3	0.92	Digoxin	sodium/potassium-transporting ATPase inhibitor
P15209	NTRK2	0.84	Entrectinib	neurotrophic tyrosine kinase receptor inhibitor
Q9CQC7	NDUFB4	0.83	Metformin Hydrochloride	mitochondrial complex 1 inhibitor, NADH/ubiquinone oxidoreductase
Q61490	ALCAM	0.63	Praluzatamab Ravtansine	CD166 antigen binding agent
Q8VDN2	ATP1A1	0.62	Digoxin	sodium/potassium-transporting ATPase inhibitor
P18572	BSG	0.59	Gavilimomab	Basigin inhibitor

a Proteins shown were matched to compounds via ChEMBL annotations in OpenTargets and include known clinically used or mechanistically validated agents. the columns list UniProt ID, gene symbol, log_2_ fold-change relative to vehicle, the associated ChEMBL-identified drug(s), and each drug’s primary mechanism of action.

## Discussion

Breast cancer remains one of the leading causes of cancer-related mortality in women worldwide,^59^ and taxane-based chemotherapeutic regimens serve as a cornerstone of clinical management. The MMTV-PyMT model recapitulates key features of human breast cancer, including progressive tumorigenesis, hormone receptor changes, metabolic rewiring, and immune activation.^24,26,60^ Increasing evidence indicates that the tumor microenvironment shapes both peripheral and CNS immune tone through changes in circulating cytokines and metabolic factors that influence susceptibility to paclitaxel neurotoxicity.^17,61,62^ This represents a largely unexplored dimension in preclinical CIPN models that lack a physiologically relevant tumor component and may contribute to mechanistic gaps in translational relevance.

In this study, we used quantitative proteomics to investigate how breast cancer modifies spinal cord responses to paclitaxel. Our findings show that cancer reshapes the spinal cord proteome in ways that may amplify paclitaxel-induced neurotoxicity, particularly across pathways regulating mitochondrial function and ion transport. These results establish insight into proteome scale changes that support the development of a translational framework for assessing PIPN in the context of tumor-driven alterations in breast cancer–bearing mice. Thus, while focused on breast cancer, this approach can be expanded in future studies to both sexes, analysis of additional neural centers of pain processing, and extended to additional cancer types, enabling a more powerful systems-level interrogation of PIPN.

### Systemic Cancer Signaling Primes the Spinal Cord for Paclitaxel Neurotoxicity

The spinal cord is a central hub in nociceptive processing, integrating peripheral sensory input from dorsal root ganglion (DRG) neurons within the dorsal horn.^63^ Neuroinflammatory activation, microglial reactivity, and neuronal hyperexcitability in the spinal cord are well-established components of CIPN,^11,12,33^ and inflammation across pain-processing regions correlates with chemotherapy-induced hypersensitivity.^8,11,12^

Recent studies highlight that the occurrence of cancer impacts neuronal vulnerability^61^ likely through systemic inflammatory cues, secretion of cytokines and chemokines, release of extracellular vesicles (EV), and production of matrix-remodeling enzymes.^16,64,65^ Neuroimmune crosstalk is known to alter synaptic transmission through neuronal and glial modulation.^66^ Tumor-derived signaling may thus sensitize the nervous system to chemotherapy exposure. Molecular adaptations to cancer occurrence are reported to have direct impacts on behaviors involving sensory tests. For example, recently a study shows that paclitaxel treated MMTV-PyMT mice exhibit a greater paw withdrawal response, in the von Frey test, than their noncancer paclitaxel treated counterparts.^61^

MMTV-PyMT tumors are characterized by extensive ECM remodeling and immune infiltration.^24^ In our proteomic results, transplantation of MMTV-PyMT cancer cells independently increased the abundance of laminin subunits and adhesion-related proteins including CADM3, CADM4, CDH13 within the spinal cord. KEGG, Reactome, and WikiPathways analyses further supported enrichment in cell organization pathways in the cancer model. Together, these findings suggest that breast cancer occurrence may prime the spinal cord through modification of ECM and cell adhesion pathwaysas well as immune cell infiltration, potentially increasing vulnerability to paclitaxel-induced injury in sensory regions.^21^

### Paclitaxel Proteomic Changes Point to Impairments in Ion Transport, Synaptic Signaling, and Mitochondrial Bioenergetics

Paclitaxel stabilizes microtubules and disrupts axonal transport, leading to mitochondrial dysfunction, a hallmark of CIPN and a major driver of sensory neuron degeneration.^12^ Our proteomic analysis indicates remodeling of proteins involved in mitochondrial and plasma membrane homeostasis, ionic gradients, and neurotransmitter cycling. These results suggest a changes in neural mechanisms for metabolic, immune, and neurotransmission activity within the spinal cord of paclitaxel-treated breast cancer-bearing mice. Markov cluster based bioinformatic analysis points to some key pathways.

(1) Synaptic transporter remodeling: The strongest paclitaxel-responsive protein cluster (Cluster 1) revealed large-scale changes in neurotransmitter transporter biology and point to cell type specific protein adaptations. Upregulated proteins included the glutamate transporters SLC1A2 (EAAT2) and SLC1A3 (EAAT1), the GABA transporters SLC6A1 (GAT1) and SLC6A11 (GAT3), the GABAergic neuron enriched cytosolic calcium buffer PVALB, and the astrocyte enriched water channel protein AQP4. These proteins regulate synaptic clearance of glutamate and GABA, astrocyte–neuron coupling, and extracellular osmolarity. Their upregulation suggests substantial remodeling of both excitatory and inhibitory synaptic tone in spinal cord neurons and the involvement of glial enriched proteins in central sensitization.^67^ These pathways are consistent with a recent report that paclitaxel triggers astrocyte-driven remodeling, including Sparcl1-dependent excitatory synapse formation, in breast tumor-bearing mice that contribute to heightened neuropathic vulnerability.^66^
(2) Cluster 2 revealed increases in mitochondrial transmembrane transport proteins most notably VDAC1, VDAC2, MICU1, and several SLC25 transporters. VDAC and MICU1 regulate mitochondrial calcium influx, and their upregulation suggests enhanced mitochondrial calcium loading, a process known to precipitate ROS generation, bioenergetic failure, and neuronal degeneration in neuropathy models.^68^ Mitochondrial calcium dysregulation has been implicated in paclitaxel-induced thermal hyperalgesia and spontaneous pain behaviors in rodents.^69,70^

(3) Paclitaxel-treated spinal cords also exhibited changes in proteins involved in ion handling (Cluster 3). These included upregulation of Na^+^/K^+^ ATPase α-subunits (ATP1A1, ATP1A2, ATP1A3), β-subunits (ATP1B1, ATP1B2), and regulatory proteins (FXYD1, FXYD7, ATP12A). Increased expression of Na^+^/K^+^ ATPase subunit proteins is consistent with compensatory hyperactivity in response to paclitaxel-induced sodium channel dysfunction and neuronal hyperexcitability.^12,71,72^ An upregulation in Na^+^/K^+^ ATPase components has been observed in other studies of neuropathic pain associated with enhanced excitatory drive.^73^
(4) Mitochondrial injury: Cluster 4 was enriched for mitochondrial Complex I proteins, including MT-ND5, NDUFB4, NDUFB6, and NDUFB9. Upregulation of these proteins suggests responses to mitochondrial stress, consistent with prior observations of ETC remodeling, ATP depletion, and mitochondrial swelling during CIPN.^16,74,75^

(5) Inflammation: Cluster 5 consists of downregulated acute phase proteins with known anti-inflammatory properties including GC, TRF, ORM2, and ITIH4. These results suggest a shift toward inflammatory signaling with the spinal cord.^76–78^ At this stage, we cannot fully exclude the possibility that some detected proteins originated from residual blood, as spinal cord tissue was not perfused prior to collection. However, we do not believe that the observed proteomic changes are primarily driven by vascular contamination, since the majority of identified proteins map to established spinal cord and neuroimmune pathways. Moreover, the inflammatory signatures observed here are highly consistent with prior rat and mouse studies demonstrating that paclitaxel induces cytokine release, immune priming, and glial activation within spinal cord tissue.^8,31–33^

Together, these findings support an integrated model of PIPN and breast cancer where cancer primes the spinal cord through ECM remodeling and immune infiltration, generating a pro-inflammatory and destabilized neural environment. Against this backdrop, paclitaxel disrupts neuronal function through ETC remodeling, mitochondrial calcium overload (VDAC1/2, MICU1), altered ion transport (ATP1A1–3, ATP1B1–2), and changes in glutamate and GABA cycling (SLC1A2, SLC1A3, SLC6A1, SLC6A11). Similar patterns of transcriptomic and proteomic dysregulation involving inflammatory and ion transport pathways have been reported in oxaliplatin-treated models of CIPN, suggesting a broader role for mitochondrial energy stress, ionic instability, and disrupted neurotransmission in chronic pain pathophysiology.^17^

### Using the Proteome to Guide the Identification of Druggable Targets for CIPN Treatment

A major outcome of proteomic analysis is the identification of signaling pathways that may serve as candidate therapeutic targets for CIPN. By leveraging an unbiased, hypothesis-generating proteomics approach, our study provides a framework for prioritizing potentially druggable proteins and pathways associated with paclitaxel-induced neuroimmune and inflammatory responses within the spinal cord. Pharmacologically actionable proteins are presented in Table 2, while Figure 8 summarizes hypothetical compound–target interactions with potential relevance to PIPN treatment.

Duloxetine, a serotonin-norepinephrine reuptake inhibitor, is the only recommended treatment option for patients with CIPN per the American Society of Clinical Oncology (ASCO) guidelines.^79,80^ Our proteomic data set highlights several druggable nodes that may serve as therapeutic targets for CIPN. Integrating our protein expression data with OpenTargets ChEMBL annotations revealed multiple FDA-approved or investigational compounds with specificity for proteins upregulated in paclitaxel-treated spinal cord.^37,38^ Several highly upregulated Complex I subunits (MT-ND5, NDUFB4, NDUFB6, NDUFB9) are known targets of metformin, which inhibits Complex I and has emerging evidence supporting mitochondrial protection in neuropathy models. Metformin has been shown to reduce mitochondrial ROS, restore ATP production, and attenuate mechanical allodynia in models of peripheral nerve injury and diabetic neuropathy, in part through AMPK activation and suppression of neuroinflammatory signaling.^81^ Its ability to normalize mitochondrial function makes it an attractive candidate for CIPN, where mitochondrial injury is a prominent mechanistic driver. While rodent studies showed that metformin prevented paclitaxel and cisplatin-induced CIPN,^82^ a recent small clinical study showed that metformin modestly reduce the incidence of CIPN in patients receiving paclitaxel chemotherapy.^83^

Upregulation of SLC6A1 (GAT1) and SLC6A9 (GlyT1) implicates tiagabine and bitopertin, respectively, as potential modulators of inhibitory neurotransmission. Tiagabine enhances synaptic GABA levels and has demonstrated analgesic properties in neuropathic pain models by dampening dorsal horn hyperexcitability and reducing spinal wind-up responses.^84^ A pilot study evaluated the potential of tiagabine hydrochloride (Gabitril) in treatment of painful sensory neuropathy however its efficacy needs to be validated in a larger study.^84^ Additional targets can broaden the therapeutic landscape further. Carbonic anhydrase CA14, inhibited by sulthiame, is involved in neuronal excitability with clinical efficacy in treating sleep disorders and a subset of epilepsy.^85^

## Conclusions

Our proteomic analysis, combined with a clinically relevant tumor-bearing model of breast cancer, provides a translational framework for identifying molecular mechanisms and therapeutic targets associated with PIPN. The present study captures proteome-scale adaptations within the spinal cord at a defined stage of disease progression, revealing alterations in neuroimmune signaling, mitochondrial function, ion transport, and neurotransmission pathways linked to pain processing. While the current work focuses on a single anatomical region and time point, future studies will expand this framework by examining additional pain-processing structures, including the DRG, across multiple stages of PIPN development and resolution. Such approaches will help define the temporal and spatial progression of proteomic adaptations underlying chemotherapy-induced neuropathic pain. Importantly, the proteomic signatures identified here provide a foundation for targeted mechanistic validation and therapeutic prioritization using complementary molecular and functional approaches. By linking dysregulated proteins to compounds with established mechanisms of action, our data set further supports a rational strategy for drug repurposing and the development of novel interventions aimed at preventing or treating PIPN in breast cancer patients.

## Supplementary Material





## Data Availability

Mass spectrometry proteomics data have been deposited to the ProteomeXchange Consortium via the PRIDE repository (http://www.ebi.ac.uk/pride/archive/) under data set identifier PXD074140.

## Notes

Figure S1. Paclitaxel reduces tumor burden in MMTV-PyMT mice. Figure S2. Principal component analysis (PCA) of spinal cord samples using proteomic data (PDF).
Table S1. (XLSX) Differentially expressed protein groups in lumbar spinal cord from MMTV-PyMT breast tumor-bearing mice compared with naïve controls, identified by quantitative LC–MS/MS proteomics. Table S2. (XLSX) Differentially expressed protein groups in lumbar spinal cord from paclitaxel-treated MMTV-PyMT breast tumor-bearing mice compared with vehicle-treated controls, identified by quantitative LC–MS/MS proteomics. Table S3. (XLSX) List of shared significantly altered proteins between PAC-Cancer to VEH-Cancer and the VEH-Cancer to VEH-NoCancer comparisons. Table S4. (XLSX) Differentially expressed protein groups in lumbar spinal cord from paclitaxel-treated naïve mice compared with vehicle-treated controls, identified by quantitative LC–MS/MS proteomics (XLSX).
