# Supplementary material for: Proteomic Adaptations in the Spinal Cord of a Breast Cancer Model of Paclitaxel-Induced Peripheral Neuropathy
Source: J Proteome Res. 2026 Jul 23;25(8):4072–85. doi: 10.1021/acs.jproteome.6c00124 (PMC13459547; doi:10.1021/acs.jproteome.6c00124)

Supplement File

Proteomic Adaptations in the Spinal Cord of a Breast Cancer Model of Paclitaxel-Induced Peripheral Neuropathy

Samantha P. Schwarting[1#], Virginia Espina[2#], Paul S. Russo[2], Sara M. Herz[3], Abrar Khan[3], Yogesh Rakholia[3], Sandra Makar[4], Paula D. Bos[4], M. Imad Damaj[3], Nadine Kabbani[1*]

[1]School of Systems Biology, George Mason University, Manassas, Virginia 20110, United States
[2]Center for Applied Proteomics and Molecular Medicine, Manassas, Virginia 20110, United States
[3]Department of Pharmacology & Toxicology, Virginia Commonwealth University, Richmond, Virginia 23298, United States
[4]Department Pathology, Massey Comprehensive Cancer Center, Virginia Commonwealth University School of Medicine, Richmond, VA 23298, United States

[#]Contributed Equally

***Corresponding Author**
Email: nkabbani@gmu.edu
Phone Number: 703-993-4406

Table of Contents

Figure S1. Paclitaxel reduces tumor burden in MMTV-PyMT mice.
Figure S2. Principal component analysis (PCA) of spinal cord samples using proteomic data.
Table S1. (XLSX) Differentially expressed protein groups in lumbar spinal cord from MMTV-PyMT breast tumor-bearing mice compared with naïve controls, identified by quantitative LC-MS/MS proteomics.
Table S2. (XLSX) Differentially expressed protein groups in lumbar spinal cord from paclitaxel-treated MMTV-PyMT breast tumor-bearing mice compared with vehicle-treated controls, identified by quantitative LC-MS/MS proteomics.
Table S3. (XLSX) List of shared significantly altered proteins between PAC-Cancer to VEH-Cancer and the VEH-Cancer to VEH-NoCancer comparisons.
Table S4. (XLSX) Differentially expressed protein groups in lumbar spinal cord from paclitaxel-treated naïve mice compared with vehicle-treated controls, identified by quantitative LC-MS/MS proteomics.

Figure S1. Paclitaxel reduces tumor burden in MMTV-PyMT mice. Longitudinal assessment of tumor volume shows paclitaxel-treated MMTV-PyMT tumor-bearing mice show significant reduction in tumor volume starting at day 18 (6 days after first paclitaxel dose).

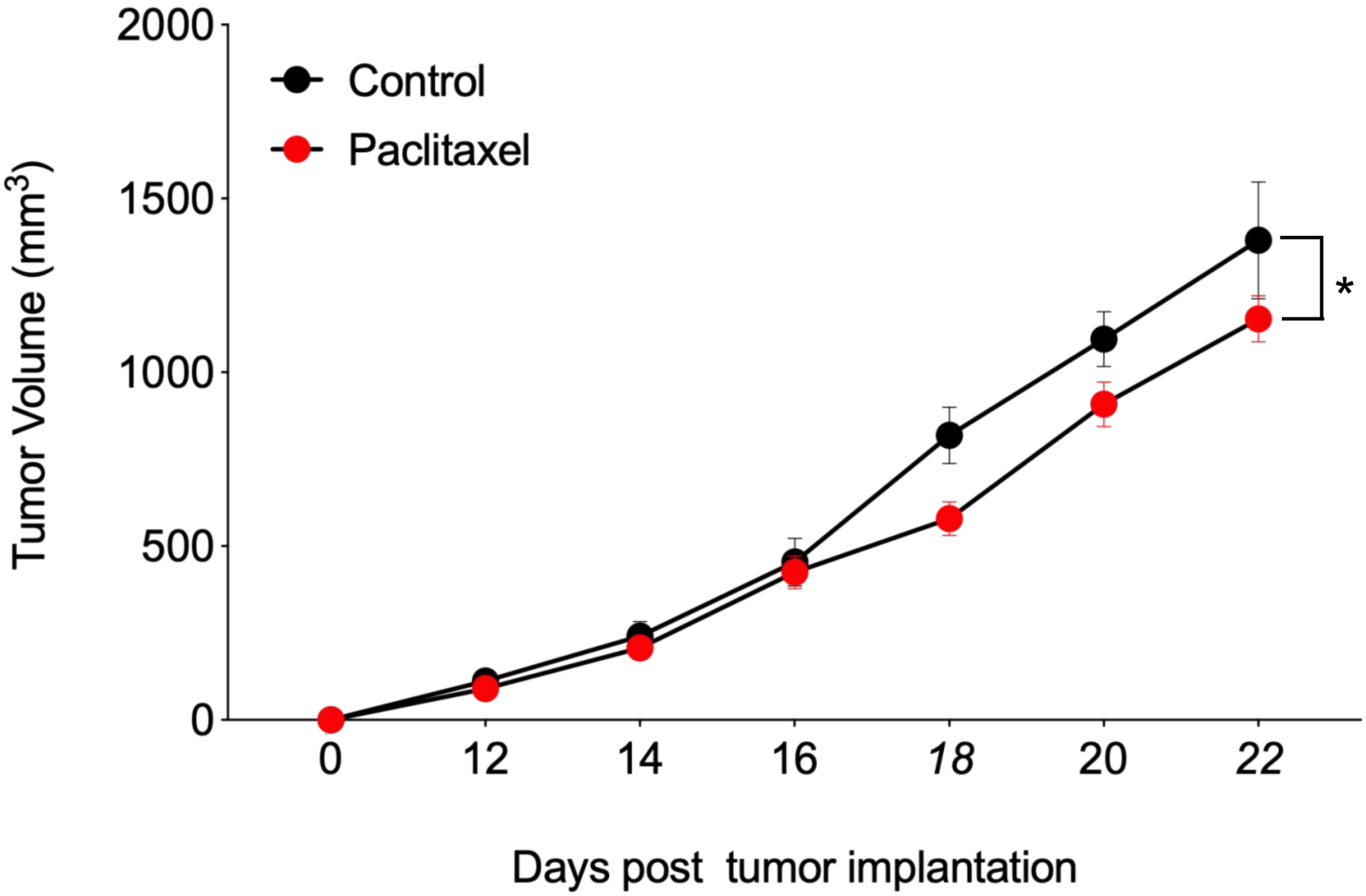

Figure S2. Principal component analysis (PCA) of spinal cord samples using proteomic data. (A) PCA of MMTV-PyMT tumor-bearing mice (pink) and naïve samples (gold) show clear separation by cancer status along the PC1 axis. (B) PCA of paclitaxel treated tumor-bearing mice (purple) and vehicle treated tumor-bearing mice samples (pink).

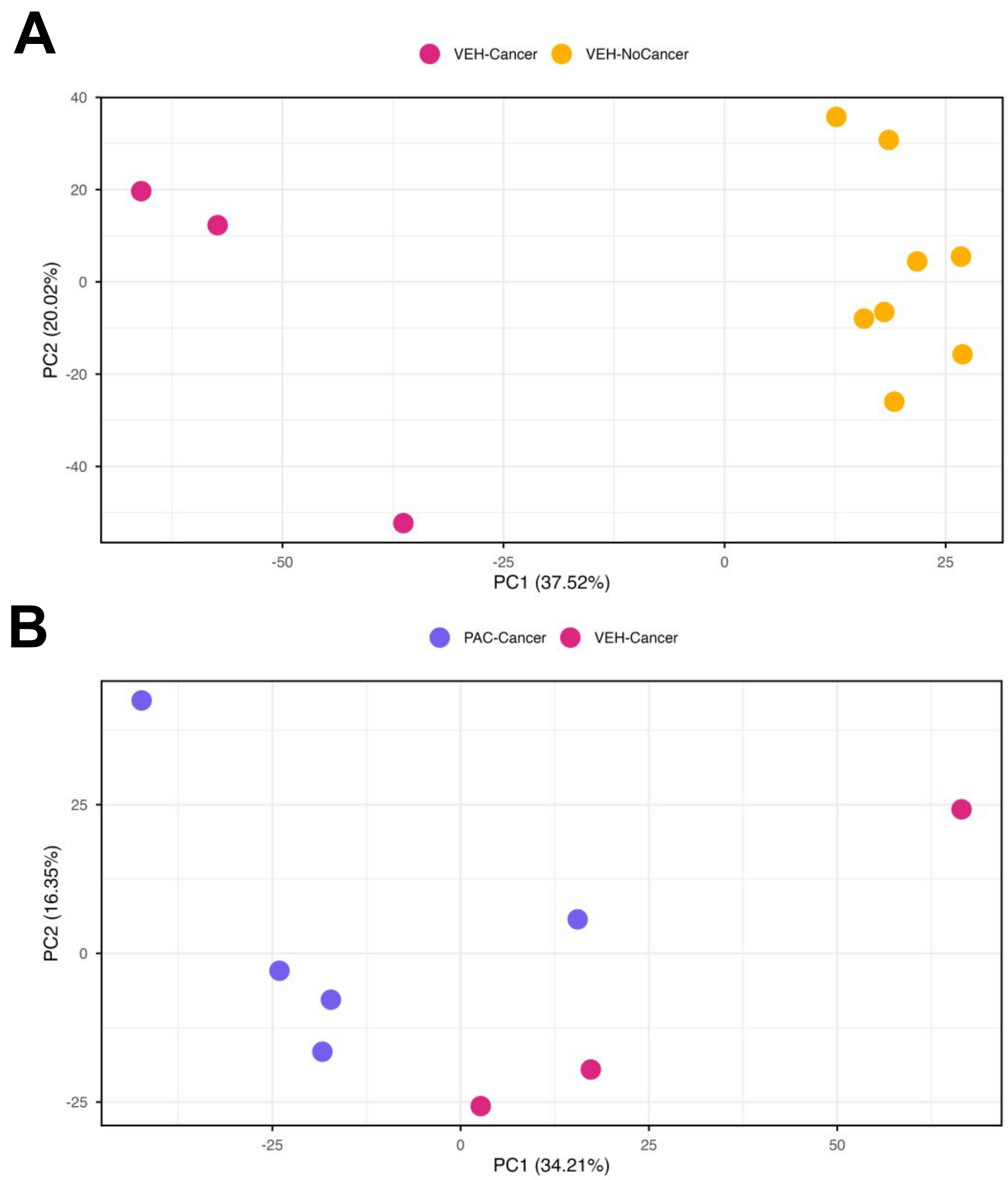

Supplement: Supplementary file 1 [file pr6c00124_si_002.pdf]
